# Natural Compounds and Autophagy: Allies Against Neurodegeneration

**DOI:** 10.3389/fcell.2020.555409

**Published:** 2020-09-22

**Authors:** Alessandra Stacchiotti, Giovanni Corsetti

**Affiliations:** ^1^Division of Anatomy and Physiopathology, Department of Clinical and Experimental Sciences, University of Brescia, Brescia, Italy; ^2^Interdepartmental University Center of Research “Adaptation and Regeneration of Tissues and Organs (ARTO),” University of Brescia, Brescia, Italy

**Keywords:** autophagy, polyphenols, alkaloids, terpenes, spermidine, trehalose, Alzheimer’s disease, Parkinson’s disease

## Abstract

Prolonging the healthy life span and limiting neurological illness are imperative goals in gerontology. Age-related neurodegeneration is progressive and leads to severe diseases affecting motility, memory, cognitive function, and social life. To date, no effective treatments are available for neurodegeneration and irreversible neuronal loss. Bioactive phytochemicals could represent a natural alternative to ensure active aging and slow onset of neurodegenerative diseases in elderly patients. Autophagy or macroautophagy is an evolutionarily conserved clearing process that is needed to remove aggregate-prone proteins and organelles in neurons and glia. It also is crucial in synaptic plasticity. Aberrant autophagy has a key role in aging and neurodegeneration. Recent evidence indicates that polyphenols like resveratrol and curcumin, flavonoids, like quercetin, polyamine, like spermidine and sugars, like trehalose, limit brain damage i*n vitro* and *in vivo*. Their common mechanism of action leads to restoration of efficient autophagy by dismantling misfolded proteins and dysfunctional mitochondria. This review focuses on the role of dietary phytochemicals as modulators of autophagy to fight Alzheimer’s and Parkinson’s diseases, fronto-temporal dementia, amyotrophic lateral sclerosis, and psychiatric disorders. Currently, most studies have involved *in vitro* or preclinical animal models, and the therapeutic use of phytochemicals in patients remains limited.

## Introduction

The mammalian central nervous system (CNS) is a crowded environment of highly specialized neurons surrounded by glial cells, fibroblasts, and pericytes, with smooth muscle cells and endothelial cells lining associated vessels (Stevens, 2003; Zeng and Sanes, 2017; Laredo et al., 2019; Matias et al., 2019). The unique post-mitotic nature of neurons, with scarce regenerative ability, means that they must have an efficient oxidative metabolism to support their specialized functions and resist cell death (Misgeld and Schwarz, 2017; Area-Gomez et al., 2018). Indeed, proper synapse function strongly depends on mitochondria, endoplasmic reticulum, lysosomes, and axonal flux of calcium ions and neurotransmitters (Wilhelm et al., 2014; Carmona-Gutierrez et al., 2016; Krols et al., 2016; Wu et al., 2017; Eisner et al., 2018). Primary inherited but also adult mitochondrial dysfunctions in neurons are not only linked to mtDNA or mtRNA changes but also to disrupted Krebs cycle, and related biochemical pathways up to reduced ATP availability. Therefore, altered morphology and signaling of crucial organelles, like mitochondria and lysosomes, dramatically initiate neurodegeneration (Lee et al., 2018; Cowan et al., 2019; Lie and Nixon, 2019) and vulnerable neurons’ death (Andreone et al., 2019).

However, autophagy, literally from ancient Greek “*self-eating*,” is an evolutionary conserved mechanism to maintain neuronal homeostasis during the development and in mature cells (Kulkarni et al., 2018). This “cleaning” pathway based on lysosomes’ activity, maintains nutrient recycling in starvation, neurotransmitter release, synaptic remodeling and pruning during development in axons and dendrites (Geronimo-Olvera and Massieu, 2019; Lieberman and Sulzer, 2019; Lieberman et al., 2019; Stavoe and Holzbaur, 2019). Recently, Tomoda et al. (2019) outlined a novel essential role of autophagy, at synaptic level, regulating information processing, memory, mood, and cognitive functions in mouse models. Moreover, Lieberman et al. (2020) discovered another essential function of autophagy in the regulation of potassium channels in neurons in the striatum of mice, necessary for excitability and motor learning. However, autophagy is not only a peculiarity of neurons but also present in astrocytes, oligodendrocytes and microglia in aging and neurodegenerative disorders (Kim et al., 2017; Plaza-Zabala et al., 2017; Belgrad et al., 2020; Wang and Xu, 2020).

Since 2006, Mizushima’s and Tanaka’s group reported dysfunctional autophagy in mice lacking fundamental autophagy genes (ATG5 and ATG7 knockout) associated to locomotor abnormalities, neuronal loss in brain and cerebellum, behavioral defects and death within 28 weeks of birth (Hara et al., 2006; Komatsu et al., 2006).

Currently, the idea of a strict interdependence between autophagy and diseases in the CNS is well defined and aberrant autophagy is associated to aging and the pathogenesis of neurodegenerative diseases characterized by abnormal proteostasis (Morimoto and Cuervo, 2014; Tanaka and Matsuda, 2014; Nikoletopoulou et al., 2015). However, in addition to macroautophagy, other degradative mechanisms are involved in the clearance of misfolded proteins in neurons such as the molecular chaperones and the ubiquitin-proteasome (Nixon, 2013; Taylor et al., 2014; Ungelenk et al., 2016; Cristofani et al., 2017; Mogk and Bukau, 2017). Recent evidence indicates that chaperones and ubiquitin-proteasome are mainly required to dismantle short-lived soluble proteins, while autophagy dismantles large misfolded aggregates in non-selective or selective manner (Chu, 2019). This last mechanism, called *aggrephagy*, implies a receptor and a substrate connection to best recognize abnormal inclusions in damaged neurons (Gatica et al., 2018). Indeed, toxic proteic aggregates impair neurotransmission, calcium flux, mitochondria activity, membrane permeability and are commonly detected in animals and post-mortem brain in patients affected by neurodegenerative diseases like Alzheimer’s (AD), Parkinson’s (PD), amyotrophic lateral sclerosis (ALS), frontotemporal dementia (FTD), and Huntington’s (HD) (Chung et al., 2018; Cheon et al., 2019; Malampati et al., 2020). However, in addition to protein aggregates, selective autophagy removes damaged organelles like mitochondria, ribosomes and the endoplasmic reticulum to maintain neuronal homeostasis (Morishita and Mizushima, 2019; Evans and Holzbaur, 2020).

Consequently, there is extensive evidence that dysfunctional autophagy and/or mitophagy have been implicated in the onset and progression of neurodegenerative and psychiatric disorders like bipolar disorder and schizophrenia (Fujikake et al., 2018; Mizushima, 2018; Bar-Yosef et al., 2019; Kuang et al., 2019; Malik et al., 2019; Palikaras and Tavernarakis, 2020).

In recent years, the belief that autophagy is a druggable target and, consequently, its modulation a promising therapeutic opportunity for CNS diseases have been reported in several authoritative reviews (Morel et al., 2017; Scrivo et al., 2018; Condello et al., 2019; Djajadikerta et al., 2019; Mputhia et al., 2019; Peng et al., 2019; Thellung et al., 2019). However, the complex pathogenesis of neurodegeneration and a better knowledge of autophagy steps and upstream signaling pathways are necessary to extend promising results obtained in animal models to patients. Remarkably, autophagy modulation seems particularly favorable in an early phase of neurodegeneration and long-term autophagy modulators without side effects are urgently required (Li et al., 2015; Menzies et al., 2017; Giampieri et al., 2019).

For all these reasons, we conceived this critical review focusing on recent studies on dietary natural products and herbs able to regulate autophagy and limit neurodegeneration *in vitro*, in rodent models and eventually in patients. Before analyzing the specific role of natural products in neurodegenerative diseases, a brief explanation of the autophagic mechanisms activated in the brain is shown below.

## Autophagic Signaling

There are three crucial types of autophagy deeply characterized in neurons (Bento-Cuesta et al., 2017) and less in glia (Strohm and Behrends, 2019), defined macroautophagy, chaperone-mediated autophagy (CMA) and endosomal microautophagy.

### Macroautophagy

Macroautophagy (simply referred as “autophagy” hereafter), the most studied dynamic mechanism of recycling macromolecules and organelles, may be beneficial or detrimental for neurons, depending on its intensity, speed (called “the autophagic flux”) and regulation (Feng et al., 2014; Button et al., 2015; Hansen et al., 2018). Indeed, there is a basal beneficial autophagy necessary for proper development to maintain life span and prolong longevity, and a detrimental excessive autophagy, called autophagic cell death or autosis (Galluzzi et al., 2018). This last type is predominant in the hippocampus, where the loss of adult stem neurons, consequent to insulin withdrawal, induces cognitive deficits (Yu et al., 2008; Jung et al., 2020). Moreover, also selected autophagy of mitochondria, called mitophagy, whenever excessive becomes detrimental leading to neuronal death, such as reported in ischemic and hypoxic events *in vitro* and in rat spinal cord (Feng et al., 2018; Yu et al., 2018). Recent studies in preclinical animal models and in post-mortem brain samples from patients indicated a strict connection between defective autophagy and mitophagy to the pathogenesis of Parkinson’s (Gao et al., 2017; Arotcarena et al., 2019; Lin et al., 2019) and Alzheimer’s disease (Chakravorthy et al., 2019; Xie et al., 2020).

However, it is essential to precisely modulate and monitor each step of the autophagy process to avoid detrimental irreversible effects instead of benefits in neurons (Mariňo et al., 2011). Therefore, we resumed below different stages of mammalian autophagy, considering that for each step it is possible to foresee a genetic or a pharmacologic regulation.

The first step of autophagy starts in the cytoplasm with the “phagophore,” a peculiar double membrane that subsequently elongates, and closes on itself to produce an “autophagosome,” filled with misfolded proteins, lipidic materials or damaged organelles. The “autophagosome” progressively matures and merges with lysosomes becoming an “autolysosome” to complete clearing. In the last final step, all cargo is dismantled by lysosomal hydrolases and eventually a new phagophore is reformed.

Different upstream machineries control the induction of mammalian autophagy and the first initiation step. The most studied are the serine/threonine protein kinase ULK1 (unc-51-like kinase 1), which forms an assembly with autophagy-related proteins 13 and 101 (Zachari and Gauley, 2017), and phosphatidylinositol 3-kinase (PI3K), essential for starting all the process (Devereaux et al., 2013). The following elongation step involves a detailed genetic program and autophagy-related proteins (ATGs), directly regulated by two pathways: the mTOR complex 1 (mTORC1) and Bcl2/Beclin 1, the mammalian ortholog of ATG6. Intriguingly, mTORC1 downregulates ULK1, so inhibiting autophagy, but may be further positively regulated by Akt or negatively by the AMP-activated protein kinase (AMPK). In this last case, autophagy is stimulated. Other kinases regulate Bcl2 by inhibiting its binding to Beclin 1 and stimulating autophagy (He and Klionsky, 2009). Furthermore, selected transcription factors influence the formation of autophagosomes, like the master transcriptional regulator of autophagy/lysosomal biogenesis (TFEB), and peroxisome proliferator-activated receptor alpha (PPAR alpha) (Fullgrabe et al., 2016). Remarkably, to treat devastating neurodegenerative syndromes many efforts have been addressed to modulate TFEB and, consequently, to restore proper autophagy (Cortes and La Spada, 2019).

Different set of ATG proteins regulate the maturation and the closure of the autophagosome, and mainly the ATG8/LC3, or microtubule-associated protein 1 light-chain 3 (LC3I), is necessary for the final formation of the autophagic vacuole. Indeed, for this step, the cytosolic LC3I become lipidated and associated with phosphatidylethanolamine to generate LC3II linked to the autophagosomal membrane (Mizushima, 2018). The final degradation of the cargo involves the fusion with lysosomes, strictly dependent on the sequestosome (SQSTM1 or p62) protein, that is a reliable marker of an effective autophagic flux (Sanchez-Martin and Komatsu, 2018).

**FIGURE 1:**
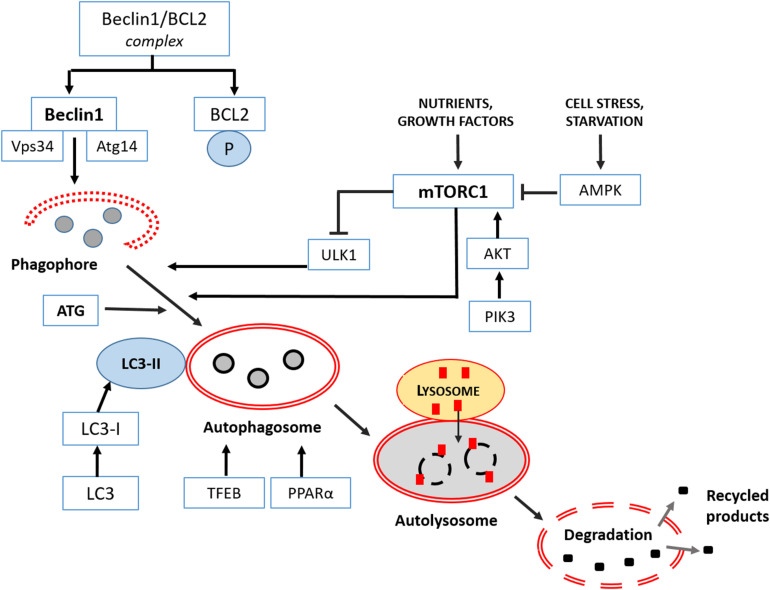
Macroautophagy signaling in the mammalian brain. It consists in three main phases: formation-elongation of a round structure, maturation then its content degradation-recycling. The first step of autophagy starts in the cytoplasm with the “phagophore,” a peculiar double membrane structure derived from ER, Golgi complex or plasmalemma that subsequently elongates, and closes on itself to produce an “autophagosome,” filled with proteins or lipidic material and damaged organelles. Then the “autophagosome” progressively matures and merges with lysosomes becoming an “autolysosome” or “amphisome”to complete clearing. In the last final step, all cargo is dismantled by acidic lysosomal hydrolases and eventually a new phagophore is reformed. Major regulators of different phases are class III PI3-K complex and Beclin 1 protein, Atg proteins cascade resulting in Atg3/Atg7 mediated LC3I into LC3II conversion as indicated and discussed in the text. Extensive details on macroautophagy machinery are reported by Feng et al. (2014).

Mitophagy, the selective autophagy of mitochondria, requires specific receptors, able to select damaged mitochondria for the removal (Gatica et al., 2018). The most studied are PTEN-kinase 1 (PINK1) and Parkin. PINK1, located in the outer mitochondrial membrane, recruits parkin, an ubiquitin ligase, from the cytoplasm to the depolarized mitochondria, making them recognizable by the autophagosome for dismantling. Altered PINK1/parkin signaling in dopaminergic neurons has been strictly associated to the pathogenesis of PD (Truban et al., 2017; Lin et al., 2019; Noda et al., 2020), and defective mitophagy is an additional hallmark of AD, FTD and ALS diseases (Cai and Jeong, 2020; Xie et al., 2020). Recent evidence indicates in autosomal recessive PD the recruitment of PINK1 to mitochondria-associated membrane (MAM) and the regulation of mitochondria-ER distance and mitophagy (Gelmetti et al., 2017). Intriguingly, an abnormal tethering and consequent disrupted mitophagy have been described in long projecting axon neurons and glia in ALS (Bernard-Marissal et al., 2018). Curiously, mice lacking PINK1 or Parkin do not present severe PD evidences like neuronal loss or locomotor dysfunctions, typical of humans, but probably due to their limited life span (Evans and Holzbaur, 2020). However, in hypoxia, different mitophagy pathways driven by novel receptors like NIX (Nip3 like protein X)/BNIP3 (Bcl2/adenovirus E1B 19 kDa protein-interacting protein 3) or FUNDC1 (FUN14 domain containing 1) are activated. Recently, in mutant dopaminergic neurons, an *in vitro* model of PD, Ryan et al. (2018) demonstrated that cardiolipin exposure in the outer mitochondrial membrane is necessary for activating mitophagy and refolding of toxic alpha synuclein. Remarkably, all above receptors joined LC3 or gamma-aminobutyric acid receptor-associated protein (GABARAP) on the autophagosome for final mitochondria dismantling (Palikaras et al., 2018). A resumptive plot indicating the progressive macroautophagy signaling and its regulation is shown in Figure 1.

### Chaperone-Mediated Autophagy (CMA)

Compared to macroautophagy, chaperone-mediated autophagy (CMA) presents three fundamental peculiarities: it removes only misfolded cytoplasmic proteins and not organelles; it does not require mature autophagosomes but only lysosomes; the recognition of aggregated proteins and the targeting to lysosomes are performed by a cytosolic chaperone HSC70 (Kaushik and Cuervo, 2018). For proper dismantling, all cytosolic proteins must contain an amino acidic sequence related to KFERQ. The complex obtained is then up-taken by the lysosome-associated membrane glycoprotein 2 (LAMP2) and degraded. Failure in CMA has been detected in PD to dismantle abnormal alpha-synuclein aggregates (a favorite CMA substrate), but alsoin AD, TD and HD to remove excessive tau protein, TAR DNA-binding protein 43 (TDP-43) and huntingtin protein, respectively (Martinez-Vicente et al., 2010; Cuervo and Wong, 2014; Wu et al., 2015; Tripathi et al., 2019). For these reasons, targeting CMA can be considered another therapeutic opportunity if autophagy is unresponsive (Xilouri et al., 2013). Intriguingly, two pathways modulate CMA in neurons: mTORC2 that inhibits LAMP2A assembly and lysosomal p38 MAPK during ER stress response (Li et al., 2019). Indeed, CMA pathway works together with other homeostatic mechanism like the ER stress response and reduced interaction between these processes results in loss of dopaminergic neurons in the *substantia nigra* in mice (Li et al., 2017). Conversely, proper restoration of both CMA and macroautophagy, by the upstream regulation of the nuclear erythroid 2-related factor 2 (Nrf2) in astrocytes, promotes effective alpha-synuclein degradation and rescue in a PD mice model (Gan et al., 2012).

### Microautophagy

This mechanism, still poorly defined in mammalians, involves the direct recruitment of cytosolic proteins containing the sequence KFERQ via Hsc70 into a membrane invagination of a late endosome or lysosome then their full degradation (Malik et al., 2019). This peculiar autophagosome-independent transfer occurred at synaptic level and was necessary for the renovation of synaptic proteins. Sato et al. (2019) reported that rapamycin, a known activator of macroautophagy, stimulated microautophagy in cells by TFEB stimulation and hypothesized its involvement in neurodegenerative diseases. The delivery of proteins to be degraded within vesicles resembled a “secretory autophagy” mechanism and intraluminal exosomes formation (Buratta et al., 2020).

## Nutraceuticals Effective in Neurodegeneration via Autophagy

Chronic neurodegenerative diseases are untreatable and few approved synthetic drugs reduced adverse symptoms but not cure them. In this discouraging scenario, oral supplementation with vegetal bioactive derivatives or the Mediterranean diet are promising to postpone the irreversible progression of AD, PD, ALS, FTD (Vauzour et al., 2017; Hornedo-Ortega et al., 2018; Pohl and Lin, 2018; Fernandez-Sanz et al., 2019).

However, considering that the relation “one-drug, one-target” for the multifactorial pathogenesis of neurodegenerative diseases is clinically unsuccessful, traditional medicinal herbs or plants with beneficial pleiotropic effects may represent a plausible preventive auxiliary therapeutic opportunity (Cummings et al., 2014; Park et al., 2018; Di Paolo et al., 2019). Plants derived compounds or “nutraceuticals” are secondary metabolites, produced by the plants to defend themselves from pathogens or adverse environmental conditions and have been proposed as complementary “herbal Medicine” to treat Alzheimer’s (AD) and Parkinson’s disease (PD) (Ahn and Jeon, 2015; Perez-Hernandez et al., 2016; Naoi et al., 2019; Renaud and Martinoli, 2019; Chiu et al., 2020).

Nutraceuticals are largely present in fruit, vegetables, cereals, herbs used in the human nutrition, with pleiotropic anti-oxidant, anti-inflammatory, glycemia-regulating properties able to preserve brain (Kennedy and Wightman, 2011; Gonzalez et al., 2019) with fewer side-effects than synthetic drugs (Georgiou et al., 2011; Forni et al., 2019). Recently, an important source of neuroprotective compounds like phytosterols, carotenoids, fucoidans and polyphenols have been characterized also in seaweeds consumed in China and Indonesia (Schepers et al., 2020). Most of them are effective anti-inflammatory and anti-oxidants and preserve dendritic spine density in hippocampal neurons. However, the harvest and purification of seaweeds are crucial to obtain bioactive compounds to use at a dosage effective for the brain activity.

Considering the pathogenetic role of aberrant autophagy in neurodegenerative diseases, emerging evidence indicates that nutraceuticals modulators of autophagy may be promising “functional foods” (Fan et al., 2017; Prieto-Dominguez et al., 2018; Xie et al., 2019; Zeng et al., 2019). Moreover, autophagy and mitophagy are involved in the removal of aggregated proteins and dysfunctional mitochondria hallmark of AD, PD, frontotemporal dementia (FTD) and Huntington disease (HD) (Menzies et al., 2017).

In the following subheadings, we discussed and commented studies published in the last decade on nutraceuticals as regulators of autophagy and their role in neurodegenerative diseases. Natural compounds have been subdivided according to their chemical structure into four categories: polyphenols, alkaloids, terpenes and terpenoids. Finally, the last subheading, entitled “Others,” is dedicated to chemically heterogeneous compounds that are emerging against neurodegeneration and aging, like the pineal indole melatonin, the disaccharide trehalose and the polyamine spermidine, a well-known caloric restriction mimetic (Kiechl et al., 2018; Lee et al., 2018; Madeo et al., 2019).

### Polyphenols

Phenolics compounds are commonly present in human diet, due to their large presence in plants. They derived from phenylalanine and contained almost one phenol ring that contributed to different subclasses like: phenols acids, flavonoids, stilbenes and lignans (Tsao, 2010). Compounds belonging to this category deeply involved to attenuate neurodegeneration are: polyphenols like resveratrol in wine and in virgin olive oil; flavanols in cocoa, tea, apples, beans; hydroxycinnamates in coffee; flavonoids in tea, apples, onions, chocolate; anthocyanins in berries (Angeloni et al., 2017; Potì et al., 2019). The effects of polyphenols on autophagy are rapidly emerging as specific to a single step of the process for each neurodegenerative syndrome (Kou and Chen, 2017).

Resveratrol (3,5,4’-trihydroxy-trans-stilbene), largely present in plants and in red wine as derivative from Vitis viniferas, regulated AMPK/mTORC1 pathway and activated the first step of autophagy to alleviate cognitive impairment in AD mice. However, dysfunctional enhanced autophagy has been reported as pathogenic hallmark of AD in dystrophic neurites with autophagic vacuoles, upregulated mTOR and reduced Beclin 1 (Pickford et al., 2008; Lipinski et al., 2010; Bordi et al., 2016). All these studies highlighted the controversial role of autophagy in AD (Castellazzi et al., 2019) and its dependence on neuronal topology and on the stage of cognitive impairment (Liu and Li, 2019).

In a recent proteomic study Lachance et al. (2019) reported in post-mortem brain reduced gene expression for autophagy kinase complex in the para-hippocampal area and hippocampus. Remarkably, the same evidence occurs in mice deprived of BECN1-PIK3C3 complex that showed memory deficits and impaired autophagy. Conversely, the activation of the nuclear receptor binding factor 2 (NRBF2), associated to PI3K complex, greatly influenced autophagy flux progression and demolition of toxic amyloid aggregates (Yang C. et al., 2017). Recently, Reddy and Oliver (2019) reported reduced autophagy and mitophagy caused by excessive amyloidosis and tau deposition in AD. Other authors reported that resveratrol is effective to reduce abnormal beta amyloid deposition in APP/PS1 mice activating AMPK (Di Meco et al., 2020) and sustaining Beclin 1 and LC3II via a Sirtuin1 signaling. Remarkably, its low bioavailability and difficulty to cross the brain blood barrier must be taken in account for its pharmacological efficacy (Salehi et al., 2018). In fact, only the trans isoform is effective in the hippocampus of AD mice by sustaining autophagy (Porquet et al., 2014). *In vitro*, in PC12 cells, resveratrol promoted autophagy via Sirtuin 1 protein and subsequent LC3I deacetylation (Deng and Mi, 2016). Resveratrol rescued ischemic damage in the rat brain during middle cerebral artery occlusion or excitotoxicity induced by glutamine (Pineda-Ramirez et al., 2020). In these models the stilbene stimulated mitophagy by activation of AMPK, Beclin 1 and LC3II conversion. Resveratrol potentiated motoneuron recovery and decreased apoptosis, after spinal cord injury in mice, through promotion of Beclin 1, LC3II and autophagy (Hu et al., 2017). Similar results were reported by Wang P. et al. (2018) in rat spinal cord injury, where resveratrol activated AMPK and autophagic flux, and consequently inhibited mTOR pathway, and *in vitro* in PC12 cells. Moreover, resveratrol induced autophagy, in dopaminergic SH-SY5Y cells challenged with rotenone via haeme oxygenase signaling (Lin et al., 2014).

As for PD mouse model triggered by methyl-4-phenyl-1, 2, 3, 6-tetrahydropyridine (MTP), resveratrol ameliorated locomotor activity and the number of dopaminergic neurons in the substantia nigra pars compacta, promoting Sirtuin 1/LC3II and reducing p62/SQSTM (Guo et al., 2016). Sirtuin 1 was crucial to maintain tolerance to neurotoxic aggregates in AD and PD by inhibiting mTOR and promoting autophagy (Lee, 2019). Finally, resveratrol restored ATG4 and stimulated LC3 II and autophagy in neuroblastoma SH-SY5Y cells expressing mutant-Huntingtin treated by dopamine (Vidoni et al., 2018).

*Curcumin*, the main active component of curry spices, and curcuminoid from *Curcuma longa* plants have been reported to induce autophagy in AD mice limiting mTOR/Akt signaling and sustaining LC3 (Wang C. et al., 2014; Shakeri et al., 2019; Voulgaropoulou et al., 2019). *Curcumin* protected *in vitro* an AD neuronal cell model (N2a/APP695swe), enhancing a retrograde axonal flux and restoring autophagy via Beclin 1 (Liang et al., 2019). Similarly, solid lipid nanoparticles driving *curcumin* stimulated CMA in human neuroblastoma cells and in mice neuronal cells challenged with toxic beta-amyloid inducing molecular chaperones and lysosomal activity (Maiti et al., 2017). Unfortunately, despite benefits in experimental animal models (Perrone et al., 2019), the clinical efficacy of curcuminoids in AD is still controversial (Zholos et al., 2019). However, due to the strong ability of curcumin to bind β-amyloid fibrils, it has been successfully adopted as fluorescent biomarker in the retinas at early stages of AD in patients and mice (Koronyo-Hamaoui et al., 2011). Intriguingly, Zhang et al. (2018) demonstrated that *curcumin* protected hippocampal neurons in amyloid treated mouse by limiting abnormal Beclin 1 and autophagosomes formation. *Curcumin* was also effective in PD *in vitro* model of dopaminergic neurons where modulated autophagy and cleaned α-synuclein aggregates (Jaroonwitchawan et al., 2017; Li et al., 2017). Moreover, a curcumin analog compound stimulated autophagy via TFEB-lysosome signaling, independently of mTOR, in murine neuroblastoma cells and *in vivo* (Song et al., 2016).

*Green tea cathechins*, mainly epigallocatechin gallate (EGCG) from *Camelia sinensis*, are diffuse phenols with recognized multiple antioxidant, anti-inflammatory and neuroprotective properties, activating beclin 1, autophagy and mitophagy as reviewed by Prasanth et al. (2019).

*Phenolic oleosides*, in particular oleuropein aglycone (OLE) from *Olea europea*, are safe antiaging, antioxidants and neuroprotective substances present in extra virgin olive oil (Casamenti et al., 2015). OLE supplementation for 3 months to the diet is able to sustain autophagy, at the last step of fusion of autophagosomes and lysosomes, so ameliorating cortical neuronal damage in transgenic AD and PD mice (Lauretti et al., 2017). Rigacci et al. (2015) demonstrated that OLE, *in vitro* to neuroblastoma cells and in AD TgRND8 mice, was able to intensify autophagy via calcium release from sarcoplasmic reticulum, activation of calmodulin-dependent kinase kinase β (CAMKKβ) and AMPK but mTOR inhibition. Intriguingly, OLE is a pleiotropic molecule able to regulate sirtuins and consequently Atg genes activation in AD mice (Cordero et al., 2018). Recently, Al Rihani et al. (2019) reported that extra virgin olive oil derivatives are able to cross the brain blood barrier, to reduce inflammation, amyloidosis and plaques in AD mice via AMPK signaling and autophagy restoration. Moreover, OLE, tested in PC12 cells exposed to a parkinsonian toxin, reduced mitochondrial damage and accelerated autophagic flux, so reducing neuronal death (Achour et al., 2016).

*Phenolic pomegranate extracts*, added *in vitro* to dopaminergic SH-SY5Y cells, upregulated autophagy and mitophagy after toxic challenge. Remarkably, the mechanism of mitophagy involved the recruitment of PINK1 and parkin to mitochondria during chemical stress (Tan et al., 2019).

*Phenolic bacosides*, active biocomponents from *Bacopa monnieri*, were effective to reduce ROS production and lipofuscin aggregation but preserved mitochondria in aged rats. Furthermore, they ameliorated cognitive ability and memory in humans in health and AD (Manap et al., 2019) even their influence on autophagy has not been studied yet.

*Flavonoids* like quercetin coupled to nanoparticles, silibinin and wogonin have been successfully used *in vitro* in SH-SY5Y neurons, and *in vivo* in rodent and human AD, where they induced autophagy by ULK1/mTOR, beclin 1 production and clearing amyloid substance (Wang D. et al., 2014; Ashrafizadeh et al., 2019b; Liu et al., 2019; Zhang et al., 2020). *Silymarin*, a lignan extracted from seeds of *Silibum marianum*, has been employed as anti-inflammatory and anti-oxidative agent in stroke by transient forebrain ischemia in rat. The neuroprotection by silymarin was due to reduced autophagic death in the CA1 region of the hippocampus (Hirayama et al., 2016). *Baicalein*, another flavonoid from *Scutellaria baicalensis*, protected rotenone treated neuroblastoma cells and mice, a well-known PD model, promoting autophagy and preventing mitochondrial damage (Kuang et al., 2017).

### Alkaloids

Alkaloids are neuroprotective agents extracted by different plants and herbs, such as Solanacee, Papaveracee, Ranuncolacee, Amaryllidacee (Hussain et al., 2018).

*Berberine*, an alkaloid derived from *Berberis* species herbs, ameliorated autophagic flux and removed tau aggregates in AD mice, recovering memory and spatial learning (Chen et al., 2020). Intriguingly, it was also effective in removal of abnormal ubiquitinated TDP-43 deposits in frontotemporal degeneration (FTD) and amyotrophic lateral sclerosis (ALS) by autophagy (Ling et al., 2013; Chang et al., 2016). Moreover, berberine attenuated neuronal damage in a spinal cord injury model in mice, by triggering autophagy in oligodendrocytes (Wang et al., 2017). Berberine has also an important anti-cancer effect in glioblastoma by activating autophagy via AMPK signaling decreased glycolytic activity and invasive potential of cells (Chang et al., 2016; Wang et al., 2016). Oral berberine enhanced life span and stimulated autophagic markers in the brain and cerebellum of transgenic N171-82Q mice, a HD model (Jiang et al., 2015).

*Caffeine*, one of the most common alkaloids in the world, modulated autophagy in SH-SY5Y neuroblastoma cells exposed to prion derived protein, so protecting them against apoptosis (Moon et al., 2014). Recently, Luan et al. (2018) demonstrated that caffeine, supplemented in drinking water for 120 days, in PD mice triggered autophagy and CMA enhancing LC3 and LAMP2 and reversing toxic α-synuclein deposits.

*Conophylline*, an alkaloid derived from *Ervatamia microphylla*, similarly stimulated autophagy *in vitro* neurons in HD and PD mimetic models (Sasazawa et al., 2015; Umezawa et al., 2018).

*Dendrobium nobile*, an alkaloid derivative from Orchidacee very common in China, has been recently demonstrated to protect hippocampal neurons exposed to β-amyloid by promoting Beclin 1 and accelerating autophagic flux (Li et al., 2017).

### Terpenes and Terpenoids

Terpenes extracted by essential aromatic oils have a recognized anti-inflammatory and antioxidant role (Quintans et al., 2019). Monotherpenes have been recently considered also modulators of autophagy (Ashrafizadeh et al., 2019a).

*Bergamot* essential oil from *Citrus bergamot* and its derived terpene, D-limonene, have been successfully added *in vitro* to human neuroblastoma cells SH-SY5Y where they induced autophagy, increasing LC3II, and accelerated autophagic flux but independently from Beclin1 (Russo et al., 2014).

*Cubeben*, a sesquiterpene from *Piper cubeba*, reduced beta amyloid toxicity *in vitro* in primary neuronal cells recovering autophagy via PI3K/AMPK signaling and inhibiting mTOR (Li et al., 2019a).

*Ginaton*, an extract from *Ginko biloba* leaves, is a mixture of terpenoids, flavonoids and organic acids with pleiotropic roles as antioxidative and neuroprotective product, mainly after 4 h after brain ischemia in stroke (Tian et al., 2017). Li et al. (2019b) induced ischemic stroke in rats, by middle cerebral artery occlusion, and treated animals with ginaton 24 h after reperfusion once a day for 14 days. Neurological symptoms ameliorated and the infarct site decreased, together with enhanced autophagic markers via AMPK and inhibition of apoptosis.

*Geraniol*, an acyclic monotherpene present in several aromatic plants, was effective to protect neurons from rotenone stress, an *in vitro* PD model, by recovering mitochondria and decreasing α-synuclein and improving autophagic flux (Rekha and Sivakamasundari, 2018).

*Cucurbitacin E*, a terpenoid phytosterol from *Ecballium elaterium* (Cucurbitacee), partially protected PC12 neurons, treated with toxins to simulate PD, but remarkably, reduced Beclin 1 autophagy, and ameliorated autophagosomes necessary for dismantling toxic deposits (Arel-Dubeau et al., 2014).

*Carotenoids*, known also as tetraterpenoids, are common bioactive pigments present in fruits and vegetables in the human diet, such as peach, watermelon, tomato, spinach, carrots, broccoli, and seaweeds with antioxidant and anti-inflammatory activities, useful against neurodegeneration (Cho et al., 2018). Fucoxanthin, a carotenoid from brown seaweeds, has been an activator of main autophagic markers, and a neuroprotector in mice model of traumatic brain injury via Nrf2 pathway (Zhang et al., 2017).

### Others

*Spermidine*, a safe polyamine present in several seeds and plant-based food, like soy and wheat germ (Muñoz-Esparza et al., 2019), prolongs lifespan in lower organisms like flies, yeast and worms, and ameliorate cognitive ability in mice and old humans (Schwarz et al., 2018). Moreover, in addition to induce autophagy by removing inhibitory acetyltransferase EP300 (Pietrocola et al., 2015), spermidine was an anti-inflammatory drug that alleviated experimental autoimmune encephalomyelitis in mice (Yang Q. et al., 2016).

*Beta-asarone* (cis-2,4,5-trimethoxy-1-allyl-phenyl), a volatile oil from *Acorus tatarinowi* herb, has been successfully tested in an AD model in PC12 neurons challenged with amyloid (Aβ42), the toxic protein in plaques. Both autophagy, the autophagic flux and mitophagy pathways were triggered and ameliorated by this compound in a dose dependent manner (Wang et al., 2020). However, if *in vitro* this product sustained Beclin 1 and LC3II signaling, the same markers were inhibited *in vivo* in double transgenic APP/PS1 mice and autophagosomes decreased in hippocampal neurons (Deng et al., 2016). Probably, *in vivo* autophagy was not the main mechanism targeted by beta-asarone able to ameliorate memory and learning in mice.

*Melatonin*, the pineal indole, also present in vegetal food, seeds and fruit, has been considered a powerful anti-inflammatory and antioxidant dietary supplement in neurodegeneration (Shukla et al., 2019). Melatonin intake reduced experimental subarachnoid hemorrhage (SAH) in rats by blocking neuronal apoptosis and abnormal autophagy just 2 h post SAH (Shi et al., 2018). Moreover, melatonin sustained Parkin/PINK1 pathway and mitophagy but inhibited inflammasome in the same animal model (Cao et al., 2017). Similarly, *in vitro* in senescent SH-SY5Y cells, melatonin was a potent autophagy inducer and an antagonist of NF-kB signaling by sirtuin 1 deacetylase (Nopparat et al., 2017).

*Trehalose*, a disaccharide present in bacteria, yeast, fungi and plants but not in vertebrates, has caught the attention as an autophagic regulator in neurodegenerative diseases (Khalifeh et al., 2019). Palmieri et al. (2017) demonstrated that trehalose, administered to a mice model of Batten disease (a neurodegenerative lysosomal disease), stimulated the clearance of toxic aggregates via activation of TFEB, the fundamental regulator of lysosomal pathway. In AD, PD and frontotemporal dementia models, the disaccharide destroyed misfolded proteins and triggered an efficient autophagic flux and lysosomal activity (He et al., 2016; Holler et al., 2016; Tien et al., 2016). Conversely, other studies on transgenic AD mice and on alpha-synuclein challenged neuroblastoma cells and primary rat cortical neurons, reported a protective activity but independent from autophagy or a block of the final step of autophagy and autolysosomes formation (Portbury et al., 2017; Yoon et al., 2017; Lee et al., 2018). A recent study on immortalized motoneurons demonstrated that *trehalose* induced TFEB nuclear translocation and autophagy and that TFEB silencing counteracted its effect (Rusmini et al., 2019). Unfortunately, *trehalose* cannot be assumed orally in humans because degraded by trehalase, an enzyme present in the gastrointestinal tract. However, recently nanolipid-trehalose conjugated have been developed as effective autophagy inducers to overcome the poor pharmacokinetics of this sugar and its efficacy at higher doses (Colombo et al., 2019).

To recapitulate, the signaling of nutraceuticals driving autophagy *in vitro* or in rodent models of CNS diseases are shown in Tables 1, 2, respectively.

**TABLE 1:** Nutraceuticals effective against neurodegeneration via autophagy *in vitro.*

**Nutraceuticals**	**Disease model**	**Dose/Signaling**	**References**
**Polyphenols**			
Resveratrol	AD-Aβ25-35 treated PC12	20 μM- Increased PARP1-SIRT1	Lin et al., 2014; Deng and Mi, 2016; Guo et al., 2016; Vidoni et al., 2018; Pineda-Ramirez et al., 2020
	PD-Rotenone treated SH-SY5Y	20 μM-Increased Heme oxygenase	
	HD-Dopamine treated SH-SY5Y	100 μM-ATG4-LC3 activity	
	Glutamate-treated neurons	30 μM-AMPK-LC3	
Pomegranate extract	PD-SH-SY5Y	300 μg/ml for 6 and 24h- TFEB activation for mitochondrial quality control.	Tan et al., 2019
Curcumin	AD-N2a/APP695swe AD-hippocampal neurons	1–10 μM-TFEB binding	Wang C. et al., 2014; Song et al., 2016; Li et al., 2017; Zhang et al., 2018; Liang et al., 2019; Zholos et al., 2019
	PD-primary neurons	50 μM-AMPK activation;10–12 M-Restored autophagic flux	
Oleuropein aglycone	AD-SH-SY5Y	50 μM for 4 h-Free Ca^2+^ flux-CAMKKβ–AMPK activation	Rigacci et al., 2015
	PD-PC12 cells	10^–12^ M for 3 h- Activated autophagic flux	Achour et al., 2016
**Flavonoids**			
Quercetin	AD-SH-SY5Y	5 mg/ml gold-palladium nanoparticles for 24 h-Enhanced autophagosomes	Ashrafizadeh et al., 2019b
Baicalein	PD-Rotenone treated SH-SY5Y	10 μM for 24 h-activated LC3	Kuang et al., 2017
**Alkaloids**			
Caffeine	Prion treated- SH-SY5Y cells	2–8 mM-LC3II induction	Moon et al., 2014
Conophylline	HD and PD-PC12 neurons	3.5 ng/ml- Enhanced autophagic flux	Umezawa et al., 2018
Dendrobine	AD-Aβ hippocampus neurons	10 ^–8^M/L for 24h-LC3 II, enhanced autophagic flux	Li et al., 2017
**Terpenes**			
D-Limonene	Starved SH-SY5Y	0.005–0.3%-LC3II and autophagic flux activation	Russo et al., 2014
Cubeben	AD-Aβ neurons	5–20 μM for 48 h-Inhibition of PI3K/Akt	Li et al., 2019a
Geraniol	PD-Rotenone treated SK-N-SH cells	100 nM for 24 h-Increased Atg5-7-12	Rekha and Sivakamasundari, 2018
Cucurbitacin E	PD- PC12 neurons	10–10 M-regulated autophagy-lysosomal pathway	Arel-Dubeau et al., 2014
**Others**			
β-Asarone	AD-PC12 neurons	24–72 μM-LC3II -Beclin 1 induction	Wang et al., 2020
Melatonin	Senescent SH-SY5Y	1 μM L-1- Beclin 1-autophagic flux activation	Nopparat et al., 2017
Trehalose	NSC34 cells	100 mM for 24 h-TFEB activation/Akt inhibition	Rusmini et al., 2019

**TABLE 2:** Nutraceuticals effective against neurodegeneration via autophagy *in vivo.*

**Nutraceuticals**	**Disease model**	**Dose/Signaling**	**References**
**Polyphenols**			
Resveratrol	AD-AβPP/PS1 mice	1% for 10 months-AMPK/Sirtuin	Porquet et al., 2014; Guo et al., 2016; Hu et al., 2017; Zhao et al., 2017; Wang P. et al., 2018; Vidoni et al., 2018; Pineda-Ramirez et al., 2020
	Brain ischemia rat	1.8 mg/kg- AMPK/Beclin1	
	Spinal cord injury rat and mice	200 mg/kg/day i.p. for 3 days-LKB1/AMPK	
	PD-MPTP mice	100 mg/kg/day for 33 days-Sirtuin 1-LC3	
Curcumin	AD-APP/PS1 mice	160–1000 ppm for 6 months-Downregulated PIK3Akt/mTOR	Wang C. et al., 2014; Song et al., 2016; Gao et al., 2017; Li et al., 2017
	Traumatic brain injury-rat	25–100 mg/kg-Enhanced Beclin 1, LC3	Achour et al., 2016; Lauretti et al., 2017; Cordero et al., 2018;
Oleuropein aglycone	AD-TgCRND8 mice PD mice	5 mg/kg diet for 8 weeks-AMPK activation, mTOR inhibition EVOO-rich diet for 6 months-Atg5-Atg7-AMPK	Rigacci et al., 2015; Al Rihani et al., 2019
**Flavonoids**			
Quercetin	AD-PD mice	5 mg/kg/day for 4 weeks	Ashrafizadeh et al., 2019b; Liu et al., 2019; Zhang et al., 2020
	Traumatic brain injury-rat	50 mg/kg i.p. for 12–24h	
Silymarin	Forebrain ischemia-rat	7 mg/kg-reduced autophagic flux	Hirayama et al., 2016
Baicalein	PD-Rotenone injected mice	100 mg/kg i.p. for 5 weeks-LC3 activation	Kuang et al., 2017
**Alkaloids**			
Berberine	AD and HD mice	40 mg/kg-Akt inhibition	Jiang et al., 2015; Wang et al., 2017.
	Spinal cord injury-rat	20 mg/kg-AMPK activation	Chen et al., 2020
Caffeine	PD mice	1 g/L for 3 months-LC3II-LAMP2A activation	Luan et al., 2018
**Terpenes**			
Ginaton	Brain ischemia mice	50 mg/kg for 14 days-AMPK activation	Tian et al., 2017; Li et al., 2019b
Carotenoids	Traumatic brain injury mice	50–200 mg/kg i.g.-Beclin 1-LC3 activation	Zhang et al., 2017
**Others**			
β-Asarone	AD-APP/PS1 mice	10 mg/kg-Beclin 1-Akt reduction	Deng et al., 2016
Melatonin	SAH rats	5–10 mg/kg i.v.-abnormal autophagy decrease	Cao et al., 2017; Shi et al., 2018
Trehalose	Batten disease mice	2% oral-TFEB activation	He et al., 2016; Holler et al., 2016; Tien et al., 2016; Palmieri et al., 2017
	AD, PD, FTD mice		

## Non-Specific Effects of Nutraceuticals in Neurodegeneration

The majority of nutraceuticals reported here has been selected for their effects on the macroautophagy machinery in experimental neurodegenerative models. However, it is important to emphasize that their activity is non specific, because the same compound has multiple roles and may act as an anti-aging, anti-apoptotic, free radicals-scavenger or anti-inflammatory drug (Howes et al., 2020). For example, *ferulic acid*, a phenolic compound commonly present in fruits and vegetables, orally administered at 80–100 mg/kg in rats 30 min before middle cerebral artery occlusion, limits ischemia reducing apoptosis and activating autophagy (Cheng et al., 2019). Conversely, *crocin*, a flavonoid from *Crocus* and *Gardenia* species, ameliorated memory and behavior in AD rat model, reducing apoptosis and citochrome c release but was ineffective on autophagic markers beclin 1/LC3 (Asadi et al., 2015). Moreover, not only a single natural principle but often mixed formulation of components may be adopted in experimental and clinical trials. For example, *sailuotong*, a mixture of saffron from *Crocus sativus*, *Ginkgo biloba*, and *Panax ginseng*, has been tested successfully in old adults, with mild cognitive impairment, to ameliorate cognitive abilities (Steiner et al., 2018). The synergistic effect of *berberine* and *curcumin* was more potent than the single compound to improve cognitive function after 3 months of treatment in AD mice (Lin et al., 2020). Extra virgin olive oil, a mixture of polyphenols, α and γ-tocopherols, added for 6 months to the diet in a taupathy mice model, alleviated synaptic activity in the hippocampus and memory impairment (Lauretti et al., 2020). Currently, more intense pharmacological and pharmacokinetic studies are required to ameliorate safety, purity, bioavailability of nutraceuticals, together with the urgent requirement of a unique worldwide regulation (Helal et al., 2019).

## Conclusion

This review focused on the regulatory role of natural dietary compounds on autophagy to postpone or alleviate neurodegeneration *in vitro* and in animal models. Unfortunately, major knowledge on the defective autophagy in humans is urgent to successfully treat patients. The importance of preventive or therapeutic autophagy regulation in neurodegenerative diseases is still debated (Bar-Yosef et al., 2019; Maiuri and Kroemer, 2019; Park et al., 2020; Suomi and Mc Williams, 2020). Negrete-Hurtado et al. (2020) reported in ATG5 KO mice brain, a non canonical role of ATG proteins and that lipidation machinery is not required for neuronal survival. Indeed, the main function of ATG-induced LC3 lipidation is to regulate microtubular dynamic in en passant boutons. According to this study, to enhance autophagy may be deleterious in forebrain axons, affecting retrograde flux and function.

Nevertheless, there is wide consensus on the efficacy of natural pleiotropic compounds able to enhance or restored insufficient autophagy in aggregated-prone proteins pathologies like AD, PD, HD in preclinical rodent models (Djajadikerta et al., 2019; Rahman et al., 2020). Remarkably, it is clear that the neuronal damage, the intensity and stage of disease greatly condition the beneficial or detrimental use of nutraceuticals and autophagy tuning in brain injury and aging (Galluzzi et al., 2017; Yessenkyzy et al., 2020). Therefore, it must be remembered that autophagy can be a double-sided process. Ferrucci et al. (2018) critically indicated that there was not a unique beneficial role of autophagy in the hypoxic brain and that may be better to inhibit autophagy to alleviate chronic ischemia. For this reason, nutraceuticals like *Leonurine*, an active extract from *Leonorus cardiaca*, inhibited ATG pathways and autophagy, so reducing neuronal damage (Liu et al., 2016).

Moreover, neurodegenerative diseases are multifactorial and many pathways must be considered (Kuang et al., 2019). Therefore, not only autophagy but also necrosis and apoptosis contributed to neuronal cell death (Leong et al., 2020). Remarkably, natural compounds able to limit neurodegeneration *in vitro* might not be effective *in vivo* and multiple products are suggested both natural and synthetic (Mazzanti and Di Giacomo, 2016). Moreover, different types of autophagy, reciprocally regulated, might concur to alleviate abnormal proteins deposition and to clear organelles in neurons (Wang H. et al., 2018; Oshima et al., 2019; Cai and Jeong, 2020). Recently, Mizushima (2018) claimed that to measure autophagic flux in humans is still impossible and consequently the direct analysis of the efficacy of natural drugs on autophagy is lacking, due to the absence of adequate quantitative methods. However, we are confident that new discoveries on autophagy tuning in the brain may confirm the utility of safe bioactive compounds and their importance to prevent or limit unavoidable neurodegeneration. Major advances in our understanding of their mechanisms of action and pharmacokinetic and nonspecific effects are necessary for success in the struggle against neurodegenerative disorders.

## Author Contributions

AS wrote the manuscript and, Tables 1, 2. GC provided revised Figure 1 and reviewed the text. Both authors conceptualized the topic, discussed the literature data, approved the final revised version of the manuscript, and ensured the accuracy of the work and intellectual content. A professional service revised the whole text in English.

## Conflict of Interest

The authors declare that the research was conducted in the absence of any commercial or financial relationships that could be construed as a potential conflict of interest.
